# Different Use of Cell Surface Glycosaminoglycans As Adherence Receptors to Corneal Cells by Gram Positive and Gram Negative Pathogens

**DOI:** 10.3389/fcimb.2016.00173

**Published:** 2016-11-30

**Authors:** Beatriz García, Jesús Merayo-Lloves, David Rodríguez, Ignacio Alcalde, Olivia García-Suárez, José F. Alfonso, Begoña Baamonde, Andrés Fernández-Vega, Fernando Vazquez, Luis M. Quirós

**Affiliations:** ^1^Ophthalmology, Vision Sciences and Advanced Therapies Research Group, Instituto Universitario Fernández-Vega, Universidad de OviedoOviedo, Spain; ^2^Departmento de Biología Funcional, Universidad de OviedoOviedo, Spain; ^3^Departamento de Bioquímica y Biología Molecular, Universidad de OviedoOviedo, Spain; ^4^Instituto Universitario de Oncología del Principado de Asturias, Universidad de OviedoOviedo, Spain; ^5^Departmento de Morfología y Biología Celular, Universidad de OviedoOviedo, Spain; ^6^Fundación de Investigación Oftalmológica, Instituto Oftalmológico Fernández-VegaOviedo, Spain; ^7^Departmento de Microbiología, Hospital Universitario Central de AsturiasOviedo, Spain

**Keywords:** glycosaminoglycans, proteoglycans, cornea, bacterial pathogenesis, bacterial keratitis

## Abstract

The epithelium of the cornea is continuously exposed to pathogens, and adhesion to epithelial cells is regarded as an essential first step in bacterial pathogenesis. In this article, the involvement of glycosaminoglycans in the adhesion of various pathogenic bacteria to corneal epithelial cells is analyzed. All microorganisms use glycosaminoglycans as receptors, but arranged in different patterns depending on the Gram-type of the bacterium. The heparan sulfate chains of syndecans are the main receptors, though other molecular species also seem to be involved, particularly in Gram-negative bacteria. Adherence is inhibited differentially by peptides, including heparin binding sequences, indicating the participation of various groups of Gram-positive, and -negative adhesins. The length of the saccharides produces a major effect, and low molecular weight chains inhibit the binding of Gram-negative microorganisms but increase the adherence of Gram-positives. Pathogen adhesion appears to occur preferentially through sulfated domains, and is very dependent on N- and 6-O-sulfation of the glucosamine residue and, to a lesser extent, 2-O sulfation of uronic acid. These data show the differential use of corneal receptors, which could facilitate the development of new anti-infective strategies.

## Introduction

Corneal infections affect around 500,000 patients globally and can lead to reduced visual acuity, irreversible scaring, and blindness (Wilhelmus, 2002; Bourcier et al., 2003; Jinno and Park, 2015). The cornea is the major refractive element of the adult eye, and consists of three layers, including a thick middle layer known as the stroma, separated by basement membranes from an external stratified epithelium and an inner layer of endothelial cells. The corneal epithelium is the interface with the environment and represents an active barrier against pathogens, commensal bacteria, toxic stimuli, and allergens (Lambiase et al., 2011). Due to the constant physical disruption of blinking and tear flow, in addition to the presence of antibacterial components, the ocular surface of healthy individuals inherently supports a small population of bacteria (Graham et al., 2007). The development of infectious processes in the cornea necessarily involves the participation of pattern-recognition receptors that allow the adherence and colonization by pathogens. These receptors may not only be involved in the anchoring of the bacteria, but also in other aspects of the infectious process such as tissue tropism, the triggering of host response, or microbial invasion (Wilson et al., 2002). Some previous studies have shown the involvement of glycosaminoglycans (GAGs) in the development of bacterial keratitis, as in the case of *Staphylococcus aureus*, responsible for 10–25% of cases and thus a leading cause of this pathology (Hayashida et al., 2011; Park and Shukla, 2013).

GAGs are linear, anionic polysaccharides that appear as part of a diverse group of glycoconjugates called proteoglycans (PGs). They are expressed ubiquitously by all mammalian cells, appearing inserted into the plasma membrane, or stored in secretory granules, as well as being secreted into the extracellular matrix (ECM). GAGs are composed of repeating disaccharides including an amino sugar, *N*-acetylglucosamine or *N*-acetylgalactosamine, and glucuronic acid or galactose. Based on core disaccharide structures, GAGs are divided into four groups: heparan sulfate (HS), chondroitin sulfate (CS), keratan sulfate, and hyaluronic acid. Most GAGs display complex heterogeneous structures resulting from the modification of the molecule by a series of interdependent enzymatic reactions, which can include N-deacetylation of *N*-acetylglucosamine, usually followed by N-sulfation, epimerization of glucuronic acid to iduronate, and diverse O-sulfations. The GAG chains are long, ranging in molecular weight from 15 to over 100 kDa, meaning the properties of GAGs tend to dominate the chemical properties of PGs (Whitelock and Iozzo, 2005; Taylor and Gallo, 2006; Schaefer and Schaefer, 2010; Iozzo and Schaefer, 2015).

A variety of normal and pathological functions have been ascribed to GAGs, including cell adhesion and migration, organization of the ECM, regulation of proliferation, differentiation and morphogenesis, cytoskeleton organization, tissue repair, inflamation, vascularization, and cancer metastasis (Whitelock and Iozzo, 2005; Taylor and Gallo, 2006; Schaefer and Schaefer, 2010; Iozzo and Schaefer, 2015). GAG functions ultimately depend on the fine structure of the chains, composed by specific sets of variably modified disaccharides, which defines binding sites for a multitude of specific ligands, including cytokines, chemokines, growth factors, enzymes and enzyme inhibitors, and ECM proteins. Cell surface GAGs can also act as coreceptors for various signaling receptors, or as endocytic receptors for the clearance of bound ligands (Whitelock and Iozzo, 2005; Taylor and Gallo, 2006; Schaefer and Schaefer, 2010; Iozzo and Schaefer, 2015). These characteristics of GAGs and PGs facilitate the specific interaction between host and pathogen during infection. Many pathogens attack host cells by binding to their GAGs, and interactions between GAGs and pathogen proteins are often the decisive factor in the initial stages of infection (Chen et al., 2008; Kamhi et al., 2013).

The purpose of this article is to analyze the role that GAGs play in the adhesion of various pathogenic bacteria to corneal epithelial cells. The study includes both Gram-positive and -negative bacteria, and aims to identify whether GAGs are involved in their binding to corneal cells, the role of each molecular species, the PG molecules involved as well as their main location (at the ECM or at the cell surface), and certain molecular aspects of interaction, particularly the influence of sulfations of the saccharide chains. Ultimately, understanding the complexity of the adhesion process would allow for the possibility of controlling bacterial infections via preventing the adhesion or invasion stages of bacterial pathogenesis.

## Materials and methods

### Materials

The following materials were purchased from the manufacturers indicated: HS, chondroitin sulfates A, B, and C (CS A, CS B, and CS C), Heparinases I and III, chondroitinase ABC, fluorescein isothiocyanate (FITC), and phospholipase C phosphatidylinositol-specific (PI-PLC) from *Bacillus cereus*, all from Sigma-Aldrich (St. Louis, MO, USA); 90% DMEM/F-12 + GlutaMAX™ [Dulbecco's Modified Eagle's medium F-12 Nutrient Mixture (Ham)], fetal bovine serum, penicillin-streptomycin, insulin, EGF (epidermal growth factor recombinant protein), and PBS (phosphate-buffered saline), from Gibco-Thermo Fischer Scientific (Waltham, MA, USA); HiTrap Desalting column and superose 12 from GE Healthcare Life Sciences (Little Chalfont, UK); Brain-Heart Infusion broth (BHI), from Pronadisa (Madrid, Spain); RNeasy Kit and RNase-Free DNase, from Qiagen (Hilden, Germany); High-Capacity cDNA Reverse Transcription Kit and PowerSYBR Green PCR Master Mix, from Applied Biosystems (Foster City, CA, USA); synthetic peptides were from Abyntek Biopharma (Derio, Spain); mouse monoclonal anti-syndecan 1 (CD138), from DakoCytomation (Carpinteria, CA, USA); and rabbit anti-syndecan 2, goat anti-syndecan 3, and rabbit anti-syndecan 4 polyclonal antibodies, from Santa Cruz Biotechnology (Santa Cruz, CA, USA).

### Bacterial strains and cell lines

The bacterial species used in this study were *S. aureus* (16491464), *Staphylococcus epidermidis* (16546925), *Streptococcus pyogenes* (16598698), *Streptococcus pneumoniae* (16171067), *Escherichia coli* (16553780), *Kebsiella pneumoniae* (16589956), *Serratia marcescens* (16583077), *Neisseria gonorrhoeae* (50264947), *Pseudomonas aeruginosa* (16531445), and *Haemophilus influenzae* (16091813), all of which were clinical isolates obtained from the Hospital Universitario Central de Asturias. All the bacteria were grown in Brain Heart Infusion (BHI broth) at 37°C in a shaking incubator, except *S. pneumoniae, N. Gonorrhoeae*, and *H. Influenzae*, which were grown in a 5% (v/v) CO_2_ atmosphere without shaking.

HCE-2 [50.B1] ATCC CRL-11135 corneal cell line was grown in Dulbecco's Modified Eagle's minimal essential medium (DMEM). The culture broth was supplemented with 10% (w/v) fetal bovine serum, 1.5 units/ml of insulin, 10 ng/ml of epidermal growth factor (EGF), and with penicillin G/streptomycin (5000 IU/ml, 5000 μg/ml). Cultures were incubated at 37°C in a 5% (v/v) CO_2_ atmosphere.

### Fluorescein labeling

FITC labeling was performed on overnight cultures washed four times with PBS and resuspended in a 0.1 mg/ml FITC solution to an A_600_ of 0.5; incubation in the dark at 37°C under agitation proceeded for 1 h and the bacterial suspensions were centrifuged, once again washed four times with PBS to eliminate the FITC excess, and resuspended in PBS to an A_600_ of 0.5.

### Adherence assays

Adhesion of the different pathogenic bacteria to HCE-2 monolayers was performed in 24-well plates to 70–90% confluence. The media was aspirated, and the cells washed twice with PBS and then blocked with 10% fetal bovine serum in PBS for 2 h at 37°C in a 5% CO_2_ atmosphere. After further washing with PBS, 100 μl of FITC-labeled bacteria in 500 μl of PBS buffer was added and the mixture incubated for 1 h at 37°C and 5% CO_2_. Next, the wells were rinsed four times with 500 μl PBS to remove any unbound bacteria. At the end of the experiment, HCE-2 cells were disaggregated with 1% SDS and the fluorescence of the pathogens attached to them was quantified in a Perkin Elmer LS55 fluorometer set at 488 nM (excitation) and 560 nM (emission). Data for the different experiments were normalized using the adhesion value without any additive or treatment, which was given the arbitrary value of 1. Assays were performed at least in triplicate and the data are expressed as the mean ± *SD*.

To analyze adhesion to the ECM, HCE-2 monolayers under the same conditions as described above were lifted with 0.5 mM EDTA in PBS. The ECM (material remaining in the wells after the cells had been lifted) was washed twice with PBS, and each well was visually inspected to ensure that no cells remained. The bacteria were then added and the assays were performed as described above.

### Inhibition of glycosaminoglycan synthesis

The cell cultures in 24-well plates at about 70% confluence were incubated in medium containing rhodamine B 50 μg/ml or genistein 30 μM (final concentration) overnight at 37°C. The cultures were washed twice with PBS, and submitted to adherence assays, as described in the previous paragraph. Efficiency of inhibition was tested by determining the concentration of GAGs in the treated cells vs. the control cells as previously described (Barbosa et al., 2003).

### Enzymatic digestion of corneal cell-surface GAGs

Hydrolysis of HS from cell cultures was achieved by incubation at 37°C for 3 h in a 5% CO_2_ atmosphere in DMEM minimal medium with a mix of 500 mU/ml (final concentration) of each of heparinase I and III. Digestion of CS chains was carried out by 3 h of incubation under the same conditions, but using 250 mU/ml (final concentration) of chondroitinase ABC. The hydrolysis of both GAGs was performed through simultaneous incubation with heparinase I and III and chondroitinase ABC under the same conditions. The reactions were stopped with two washes in PBS buffer and the cell cultures were immediately submerged in DMEM and adherence assays with pathogens carried out as described above. The efficiency of digestions was established by determining the concentration of GAGs in treated cells vs. control cells as previously described (Barbosa et al., 2003).

### Adherence inhibition assays

The experiments examining the effect of GAGs on adherence interference were performed through the addition of either HS, CS A, CS B, and CS C, or a mixture of all four, at concentrations ranging between 0.01 and 200 nM to the labeled bacteria before their addition to the monolayers. However, the assays comparing HS of high and low molecular weight (MW), and those examining the differences between poorly and highly sulfated HS domains used a concentration of 10 nM.

The effect of peptides which include consensus heparin binding sequences on adherence interference experiments was initially calibrated using the peptides QKKFKN and FKKKYGKS at concentrations ranging between 1 and 500 nM, and measuring the interference they produced on the binding of *Pseudomonas aeruginosa* and *Stapylococcus aureus*. Based on these data, the effect on all pathogens was analyzed at a concentration of 20 nM of each peptide. The peptides were added to the monolayers before the addition of the labeled bacteria.

### Molecular size fractionation of HS

Molecular size fractionation of HS was carried out using size-exclusion chromatography. One hundred microliters of a 1 mg/ml HS solution including 10 nCi of [^3^H]HS were applied to a superose 12 column connected to an FPLC system (GE), and eluted with 50 mM of pH 8.0 Tris-HCl buffer and 150 mM of NaCl at a flow rate of 1 ml/min. One milliliter fractions were collected, and HS eluting from the column was determined by measuring radioactivity in aliquots of 200 μl of each fraction. Fractions including molecules of either high or low MW were pooled separately and then precipitated with 85% ethanol for 2 h at −80°C.

### Obtaining of highly and poorly sulfated domains from HS

Highly sulfated HS domains were obtained by digestion of HS chains with heparinase III, and poorly sulfated HS domains were obtained by digestion with heparinase I. In both cases 18 μg HS were digested with 100 mU enzime in a 200 μl total volume of sodium acetate buffer 100 mM pH 6.8 including CaCl2 10 mM.

### RNA isolation, cDNA synthesis, and qRT-PCR reactions

RNA was isolated from corneal cells using the RNeasy kit following the manufacturer's specifications. Samples were subjected to treatment with RNase-free DNase during the purification process itself. The concentration of RNA obtained was determined spectrophotometrically by measuring absorbance using a Picodrop Microliter UV/Vis pectrophotometer (Picodrop Limited, UK).

cDNA synthesis was carried out using the High Capacity cDNA Transcription Kit following the manufacturer's specifications. The reaction products were cleaned using the PCR Clean-Up GenElute kit following the manufacturer's instructions.

qRT-PCR reactions were carried out as previously described (Fernández-Vega et al., 2013), and actin was used as a control gene to compare run variation and to normalize individual gene expression. The expression values of the genes were calculated as 2^−ΔCt^ (relative to actin as the housekeeping gene).

### Antibody inhibition assays

Antibody inhibition assays were carried out in 24-well plates and grown to 80% confluence. The media was aspirated, and the cells were washed twice with PBS and then blocked with 10% fetal bovine serum in PBS for 2 h at 37°C in a 5% CO_2_ atmosphere. After further washing with PBS, anti syndecan-1, anti syndecan-2, anti syndecan-3, or anti syndecan-4, or a mixture of all four, in PBS were added and incubated for 1 h. All the antibodies were diluted 1:100, except anti-SDC2, which was diluted 1:250. After the treatments, adherence assays were performed as previously indicated.

### Enzymatic removal of glypicans using phospholipase-C

PI-PLC was used to enzymatically remove glypicans from the cell membrane. Cells were grown in 24-well plates to 80% confluence. After washing with PBS, the cells were incubated in the absence or presence of 80 mU/ml PI-PLC for 40 min at 37°C in a 5% CO_2_ atmosphere. After the treatment, adherence assays were performed as detailed previously.

### RNA interference

Human lentiviral constructs for NDST1 (TRCN0000008648), HS2OST1 (TRCN0000034936), and HS6ST1 (TRCN0000034564) were purchased from Thermo Fischer Scientific. Human lentiviral constructs for NDST1 and HS6ST1 were selected to include sequences shared by NDST2 and HS6ST2 respectively. Lentiviral particles were produced by cotransfection of 293T cells with the shRNA plasmid and packaging vectors pMD2.G and psPAX2 (gifted by Didier Trono; Addgene plasmids # 12259 and #12260, respectively) according to the vendor's instructions. Knockdown efficiency was determined by qRT-PCR analysis as previously described (Fernández-Vega et al., 2013).

### Statistical analysis

All analyses were performed using the Statistics for Windows program (Statsoft Inc; Tulsa, OK). Mean values between two samples were compared using the Mann-Whitney *U*-test, and between multiple samples using the Kruskal-Wallis test. *P* < 0.05 was accepted as significant. All data are presented as means ± standard error.

## Results

### GAGs are differentially involved in the binding of gram-positive and gram-negative pathogens to corneal epithelial cells

**Figure 1:**
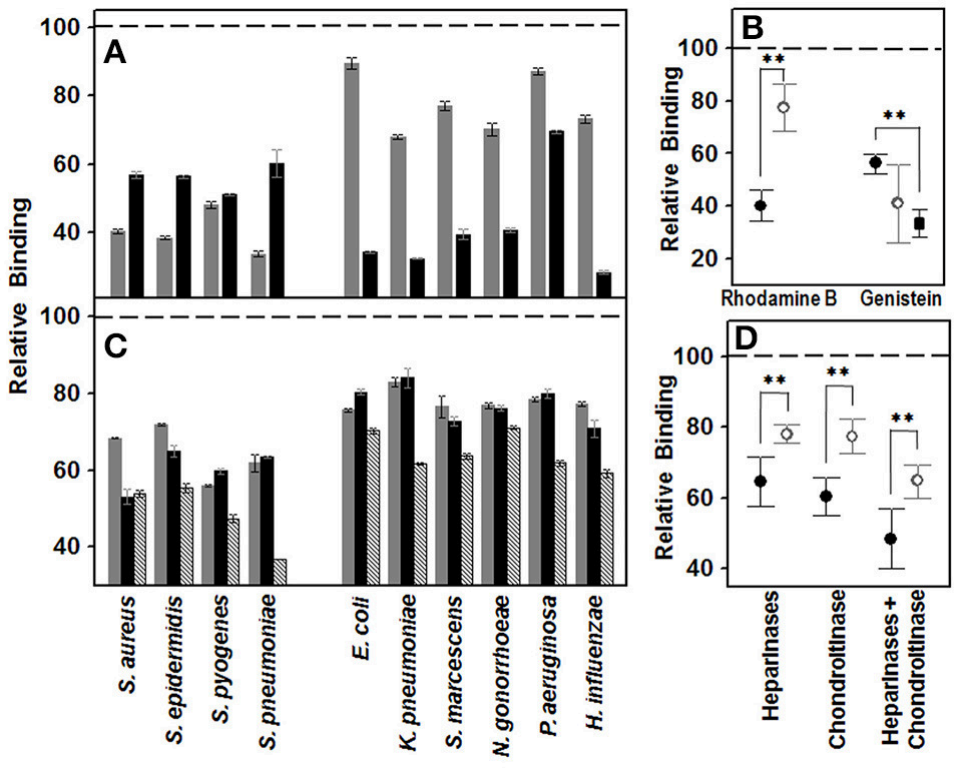
**Effect of decrease in cell GAGs on pathogen adhesion to corneal epithelial cells**. **(A,B)** Effect of inhibition of GAG biosynthesis. **(A)** Inhibition of bacterial attachment to HCE-2 cells treated with rhodamine B (gray bars) and genistein (black bars). **(B)** Differences in adherence to HCE-2 cells treated with rhodamine B or genistein between Gram-positive (●) and Gram-negative (○) bacteria. Gram-negative data for binding to genistein-treated cells are represented including or excluding (■) *Pseudomonas*. **(C,D)** Effect of the pre-treatment of HCE-2 cell cultures with GAG lyases. **(C)** Inhibition of bacterial attachment to HCE-2 cells treated with heparinases I and III (gray bars), chondroitinase ABC (black bars) or heparinases + chondroitinase (striped bars). **(D)** Adherence differences of Gram-positive (●) and Gram-negative (○) bacteria to HCE-2 cells treated with heparinases I and III, chondroitinase ABC or heparinases + chondroitinase. Data were normalized using the adhesion values of bacteria to non-treated cells, which was given the arbitrary value of 1. The spreads represent the standard deviations. Statistically significant differences are denoted by ^**^, which indicates *p* < 0.01.

To study whether GAGs mediate the binding of different common corneal pathogens, including both Gram-positive and Gram-negative bacteria, to corneal epithelial cells, their biosynthesis was inhibited using either rhodamine B or genistein. The results showed a decrease in the adherence of all the bacteria investigated, suggesting that GAGs are involved in a general way in the binding of pathogenic microorganisms to the corneal epithelium (Figure 1A). However, the observed effect was highly dependent on two factors: the inhibitor molecule used and the Gram nature of the bacterium analyzed. Rhodamine B diminished the binding of Gram-positive bacteria by 60% (±5.9), while Gram-negative binding was decreased by only 23% (±8.9). Moreover, GAG-devoid cells, obtained through treatment with genistein, reduced Gram-positive and Gram-negative bacteria binding by 44 (±3.7) and 60% (±14.8), respectively (Figure 1B); However the peculiar behavior of *P. aeruginosa*, whose binding decreased by only about 13%, demonstrates the high deviation of the result obtained for the Gram-negatives, to the extent that the exclusion of this microorganism results in a reduction of 65% (±5). Statistical analysis showed significant values for rhodamine B (*p* < 0.01) and a close to significant effect for genistein (*p* = 0.08, *p* < 0.01 if *Pseudomonas* is excluded), suggesting that inhibition by both molecules differentially affects the binding of microorganisms depending on their Gram nature. What is more, the two molecules produce opposite effects, such that inhibition by rhodamine strongly affects binding of Gram-positive bacteria, while genistein's effect is more intense with Gram-negatives (Figure 1B).

To investigate further the adherence of pathogens to the GAGs, corneal cell surface GAGs were removed by digestion with bacterial lyases, and the effect of this on the binding of the different bacteria was determined. Treatment with heparinase I and III or chondroitinase ABC reduced the adherence of the pathogens in a similar manner, with no statistically significant differences (Figure 1C). However, there were differences when the effects on either Gram-positive or Gram-negative pathogens were analyzed separately; treatment with heparinases or chondroitinase reduced the adherence of Gram-positive bacteria 35.5 (±7.05) and 40% (±5.2), respectively, while the reduction for Gram-negatives were 22 (±2.6) and 22.5% (±4.9); the differences being statistically significant for both treatments (*p* < 0.01; Figure 1D). In the combined digestion employing both heparinases and chondroitinase, adherence was significantly reduced compared to in digestions using a single enzyme (*p* < 0.05 in all cases), and statistically significant differences between Gram-positive and Gram-negative were also detected (*p* < 0.01l Figure 1D).

### Distinct GAG species are differentially involved in the binding of pathogens to corneal epithelial cells

**Figure 2:**
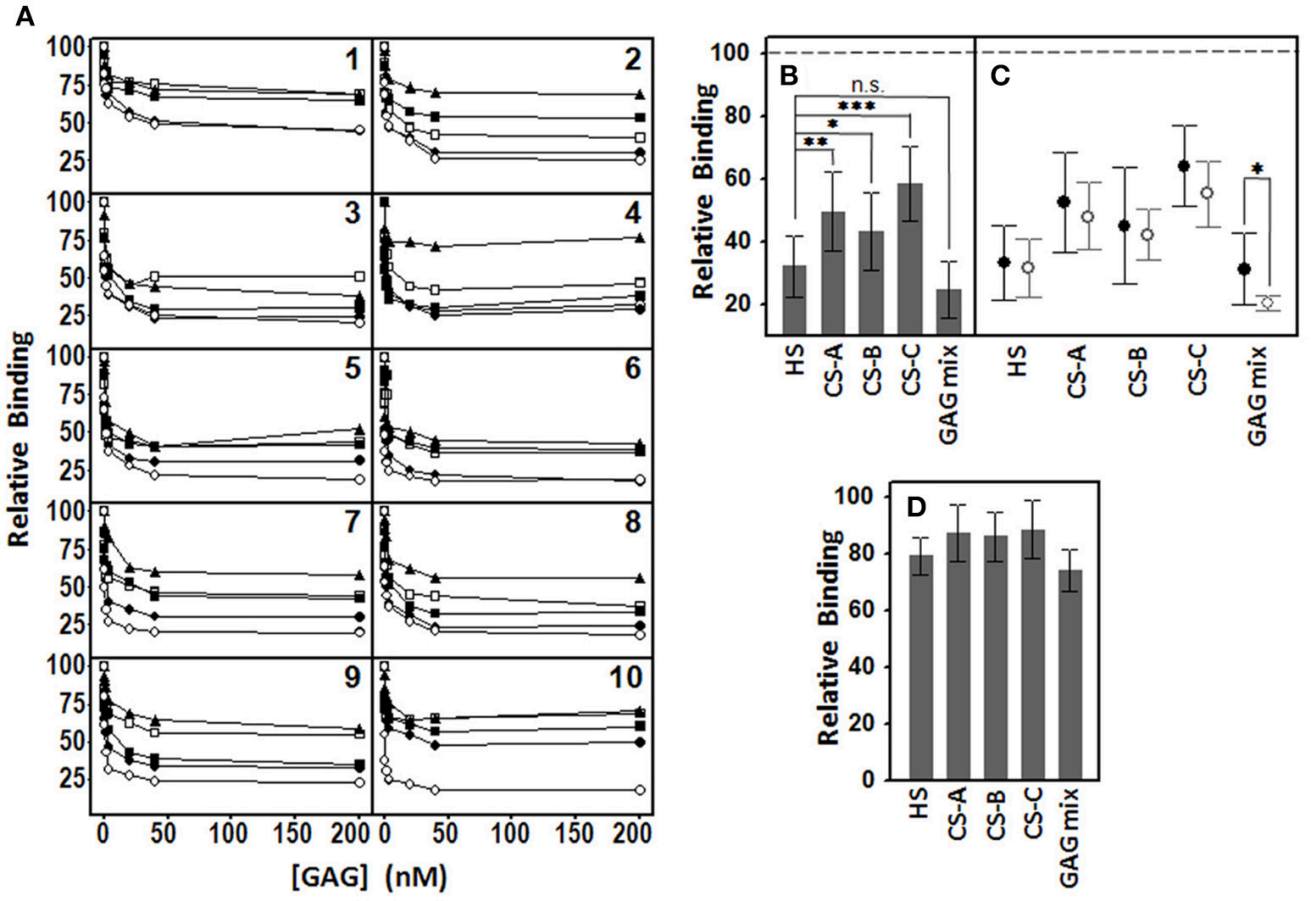
**Inhibition of pathogen attachment to corneal epithelial cells by the presence of different GAGs**. **(A)** Adhesion of *Staphyloccocus aureus* (1), *Staphyloccocus epidermidis* (2), *Streptoccocus pyogenes* (3), *Streptococcus pneumoniae* (4), *Escherichia coli* (5), *Klebsiella pneumoniae* (6), *Serratia marcescens* (7), *Neisseria gonorrhoeae* (8), *Pseudomonas aeruginosa* (9), and *Haemophilus influenzae* (10) in the presence of different concentrations of HS (●), CS A (□), CS B (■), CS C (▲), and a mixture of all GAGs (○). **(B)** Comparative effect of the different individual GAG species and the mixture of them all on bacterial adherence to corneal cells. **(C)** Comparative effect of the different GAG species and the mixture on adherence of Gram-positive (●) and Gram-negative (○) bacteria. **(D)** Comparative effect of the different individual GAG species and the mixture (40 nM) on bacterial adherence to ECM. The spreads represent the standard deviations. Statistically significant differences are denoted by ^***^, ^**^, and ^*^, which indicate *p* < 0.001, *p* < 0.01, and *p* < 0.05, respectively and n.s. indicates that differences were not significant.

To analyze the role that different species of GAGs play in the adhesion of pathogens to corneal cells, adherence interference experiments were performed using HS, CS A, CS B, CS C, and a mixture of all four, as described in the Material and Methods section. In all cases, the presence of GAG molecules decreased the adherence of bacteria to corneal cells in a dose dependent manner (Figure 2A). Furthermore, the effect was dependent on the particular species of GAG used. In all cases, HS was the most effective interfering molecule, followed by CS B, CS A and, lastly, CS C, except in the case of the two species of *Streptococcus* examined, where CS A showed a greater effect than CS B. When a mixture of all GAGs was used, the effect was similar to that obtained using HS alone, except in the cases of *E. coli, S. marcescens, P. aeruginosa*, and *H. influenzae*, which the effect of the combined treatment was greater (Figure 2A).

Taken together, the results evidence that both, HS and the mixture of GAGs, have the highest inhibitory effect, although the differences were not statistically significant (*p* = 0.1); nevertheless, the two treatments did display differences when compared to other species analyzed (*p* = 0.002, 0.04, and < 0.001 for HS compared to CS A, CS B, and CS C, respectively; Figure 2B). When the inhibitory effect of the different GAGs was analyzed separately for Gram-positive and Gram-negative bacteria, no significant differences was detected except in the case of the mixture of all the species (*p* = 0.049; Figure 2C).

To analyze whether pathogens adhere primarily to corneal epithelial cells or to the ECM, we prepared wells by detaching the cells with EDTA, as described in Material and Methods. The values of the adhesion of bacteria to the ECM were much lower than those observed for cells (usually below 5%). Adherence interference experiments carried out using the different GAG species at 40 nM showed that the adherence of bacteria decreased by around 10% with each of the CS types, by 21% for HS and by 26% for the GAG mixture, all much lower than the values obtained for the inhibition of adhesion to cells (Figure 2D).

### HS molecules are differentially involved in the binding of pathogens to corneal epithelial cells depending on their size and sulfated domains

**Figure 3:**
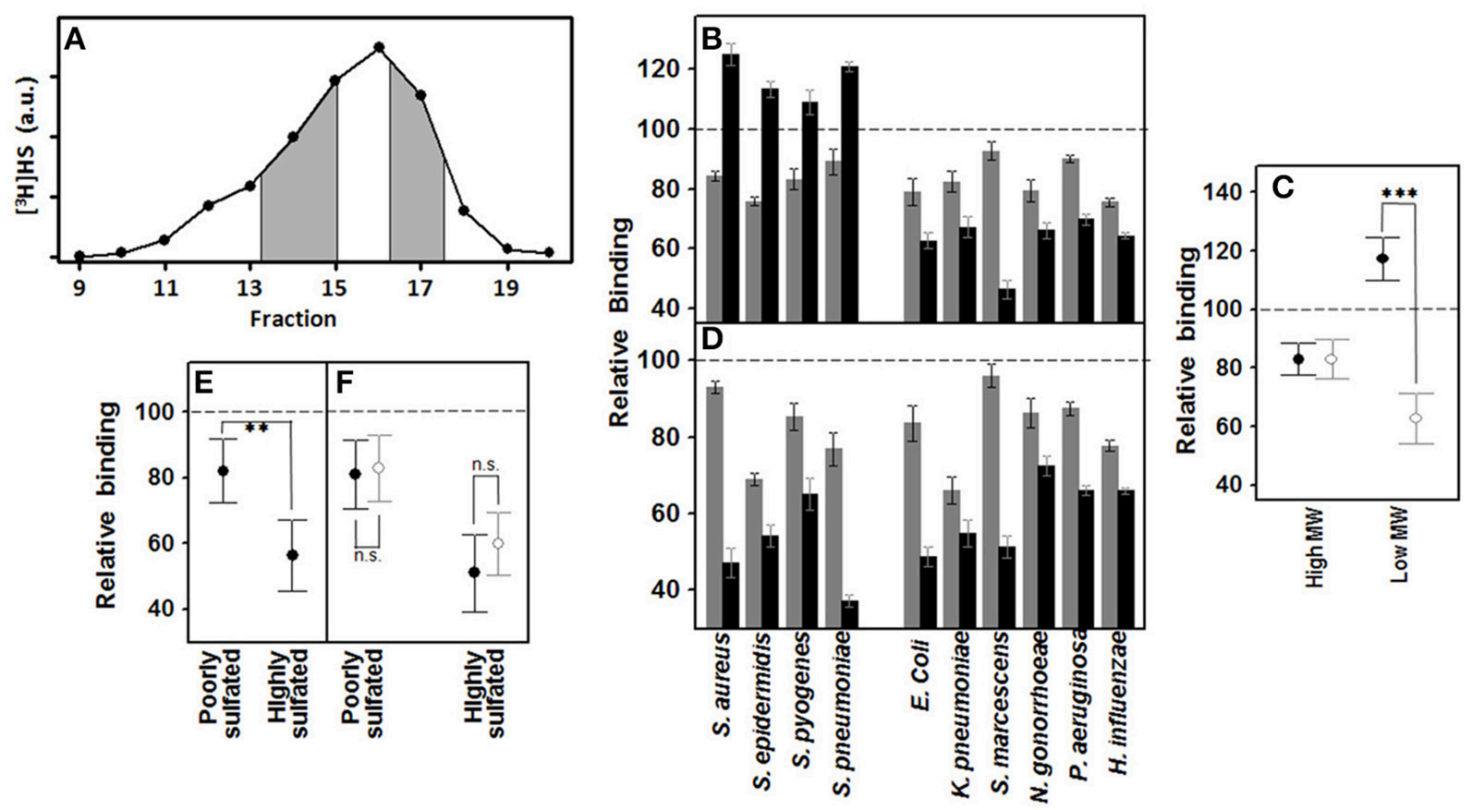
**Effect of the HS chain size and sulfation of domains on pathogen attachment to corneal epithelial cells**. **(A–C)** Effect of high and low MW HS on pathogen attachment to corneal epithelial cells. **(A)** Size exclusion chromatography of HS; fractions collected, corresponding to areas of both high and low MW, are shaded. **(B)** Effect on attachment of pathogens to HCE-2 cells: gray bars indicating high MW and black bars, low MW. **(C)** Adherence differences of Gram-positive and Gram-negative bacteria in the presence of high and low MW HS. **(D–F)** Effect of highly and poorly sulfated HS domains on pathogen attachment to corneal epithelial cells. **(D)** Effect on attachment of corneal pathogens: gray bars represent NA-domains and black bars, NS-domains. **(E)** Comparative effect of NA- and NS-domains on bacterial adherence to corneal cells. **(F)** Adherence differences of Gram-positive (●) and Gram-negative (○) bacteria in the presence of HS NA- and NS-domains. The spreads represent the standard deviations. Statistically significant differences are denoted by ^***^, and ^**^, which indicate *p* < 0.001, and *p* < 0.01, respectively. n.s. indicates that differences were not significant.

Using size exclusion chromatography, HS was separated into fractions of low (< 10 kDa) and high (>10 kDa) MW (Figure 3A). The effect of both fractions on the binding of pathogens to corneal cells was analyzed by adherence interference experiments. The results showed that high MW HS decreased the adherence of bacteria around 20%, and this value was independent of the Gram type of the microorganism (Figures 3B,C). Interestingly though, low MW HS did display different, and opposite, effects depending on whether bacteria were Gram-negative or –positive: the former diminishing their adhesion about 40%, and the latter around 20% relative to controls (Figures 3B,C), and this difference was statistically significant (*p* < 0.001).

HS chains are composed of highly sulfated NS-domains interspaced with poorly sulfated NA-domains. These two domain types were isolated by treatment with specific heparinases, and then used to carry out adherence interference experiments (Figure 3D). NS-domains in general were found to be twice as effective in terms of inhibiting the binding of the bacteria (*p* < 0.01; Figure 3E); however, there were no significant differences between the effect produced on Gram-positive or Gram-negative bacteria by the two types of domain (Figure 3F).

### Differential interference of peptides with heparin binding sequences in the binding of gram-positive and gram-negative pathogens to corneal epithelial cells

**Figure 4:**
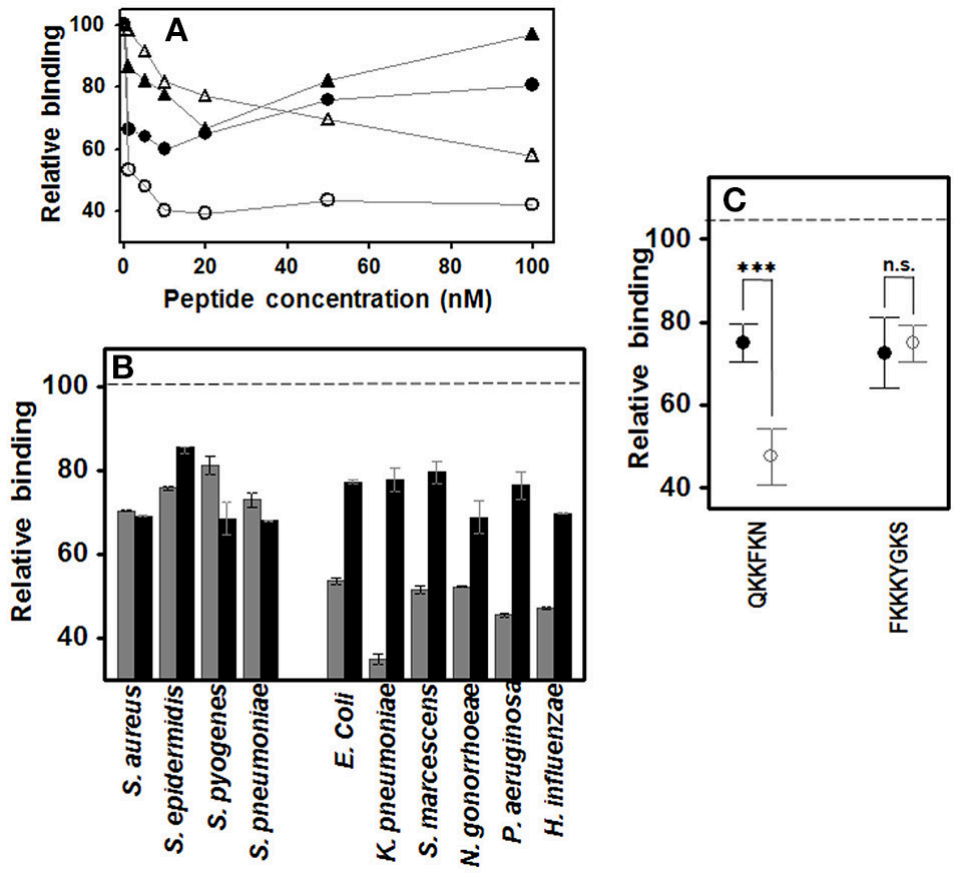
**Effect of peptides with heparin binding sequences on pathogen attachment to corneal epithelial cells**. **(A)** Effect of peptide QKKFKN on adherence of *S. aureus* (●) and *P. aeruginosa* (○), and effect of peptide FKKKYGKS on adherence of *S. aureus* (▲) and *P. aeruginosa* (Δ). **(B)** Effect on pathogen attachment to corneal epithelial cells; gray bars: QKKFKN 20 nM; black bars: FKKKYGKS 20 nM. **(C)** Adherence differences of Gram-positive (●) and Gram-negative (○) bacteria in the presence of the short or the long peptide. Statistically significant differences are denoted by ^***^, which indicate *p* < 0.001. n.s., not significant.

Two peptides were designed which included consensus heparin binding sequences of different lengths, the shorter being QKKFKN, and the longer, FKKKYGKS. The effect of the concentration of the two peptides on bacterial adherence to HCE-2 cells was determined using the Gram-positive *S. aureus* and the Gram-negative *P. aeruginosa*. In the case of *S. aureus*, both peptides decreased adherence in a dose-dependent manner up to concentrations of 10–20 nM, their effects diminishing at higher concentrations. In contrast, *P. aeruginosa* decreased its adherence in the presence of the short peptide up to 20 nM, while the longer peptide produced progressive interference, more noticeable at higher concentrations (Figure 4A). On the basis of these data, subsequent experiments were carried out at a concentration of 20 nM for both peptides.

Results showed that the longer peptide decreased adherence by about 20–30% for all the bacteria analyzed (Figure 4B). However, the shorter peptide had a similar effect on Gram-positive bacteria, but decreased the adherence of Gram-negative bacteria about 50–60%, the difference between the two groups being statistically significant (*p* < 0.001; Figure 4C).

### Involvement of cell-surface HSPGs in the binding of pathogens to corneal epithelial cells

**Figure 5:**
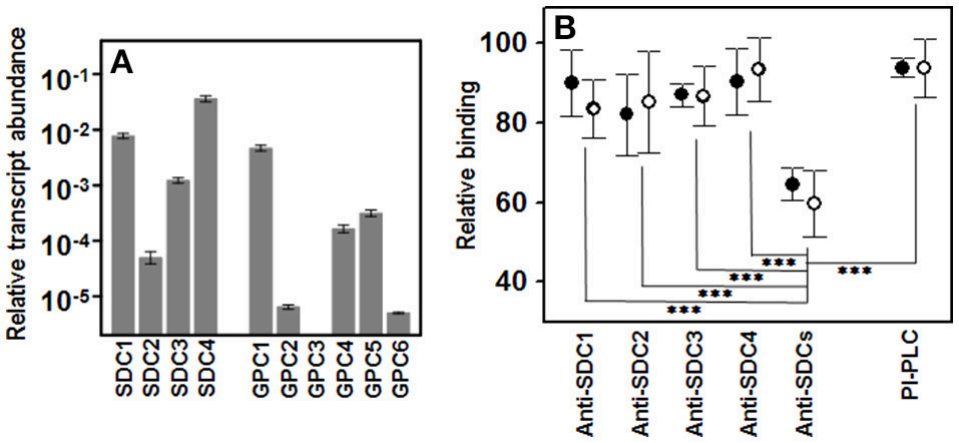
**Involvement of cell surface HSPGs in the binding of pathogens to corneal epithelial cells**. **(A)** Differential transcription of cell surface HSPGs in HCE-2 cells; values on the Y-axis are represented on a logarithmic scale. **(B)** Relative adherence differences of Gram-positive (●) and Gram-negative (○) bacteria after treatment with anti-SDC antibodies or with PI-PLC. The spreads represent the standard deviations. Statistically significant differences are denoted by ^***^, which indicates *p* < 0.001.

To find out which species of HSPGs were present on the membrane of the HCE-2 cells, the transcription levels of all the isoforms of syndecans and glypicans were analyzed by qRT-PCR reactions. The results showed the existence of transcripts for each of the four syndecan isoforms, although their respective levels varied in total by around three orders of magnitude, SDC4 and SDC1 being the most abundant, and SDC2 the most scarce (Figure 5A). In contrast, transcripts were not detected for all isoforms of glypicans and some appeared at only very low levels, GPC1 being the most abundant species (Figure 5A).

To investigate the role of glypicans in the binding of pathogens, they were removed from the cell surface using PI-PLC, an enzyme that cleaves their glycosylphosphatidylinositol-anchor. The results showed that this treatment only slightly decreased bacterial adherence, and no differences between Gram-positive and Gram- negative were detected (Figure 5B).

Since the four isoforms of syndecans were all transcribed in HCE-2 cells, their individual involvement in adherence was analyzed by blocking them using specific antibodies for each species in separate experiments. The results obtained were variable and, curiously, were not related to the apparent levels of SDC isoforms derived from their transcription levels (Figure 5B). Interestingly, when antibodies against the four isoforms were used simultaneously in the experiment, adherence decreased sharply, irrespective of the Gram nature of the pathogen (Figure 5B).

### Influence of specific N- and O- sulfations on pathogen adherence to corneal epithelial cells

**Figure 6:**
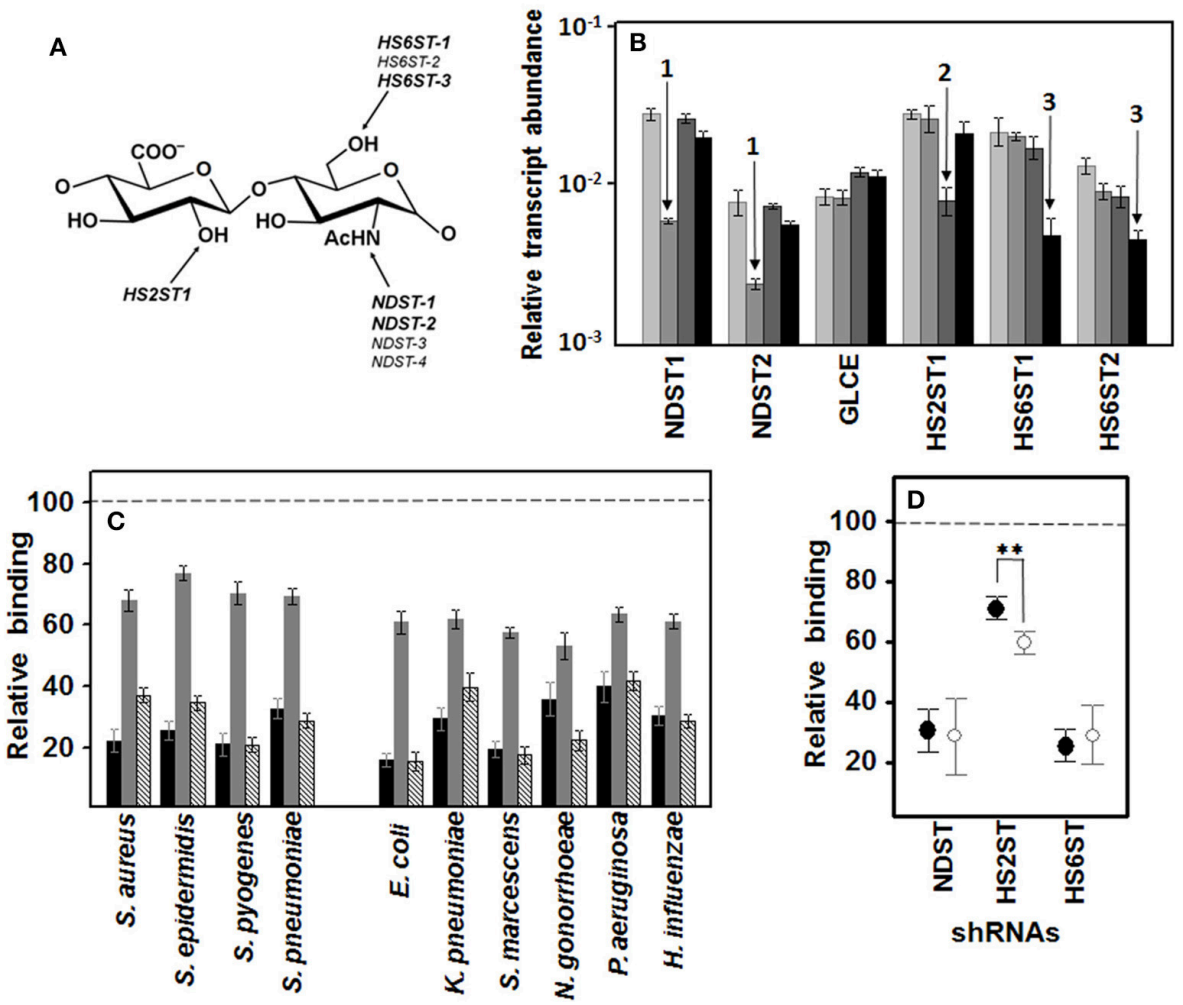
**Influence of specific N- and O- sulfations on pathogen adherence to corneal epithelial cells**. **(A)** Structure of an HS disaccharide unit showing the genes involved in more common sulfations. Genes whose transcription can be detected in corneal epithelial cells are highlighted. **(B)** Differential transcription of the genes encoding enzymes involved in HS sulfation in HCE-2 cells. The bars with increasingly darker colors indicate the control, and the clones in which N- sulfation, 2-O- sulfation and 6-O-sulfation were silenced, respectively. The arrows indicate downregulation of NDST (1), HS2ST1 (2), and HS6STs (3). NDST1 and -2: N-deacetylase/N-sulfotransferase 1 and 2; GLCE: C5-glucuronate epimerase; HS2ST1: HS 2-O-sulfotransferase; HS6ST1 and -2: HS 6-O-sulfotransferase 1 and 2. **(C)** Inhibition of bacterial attachment to HCE-2 cells with silencing of the genes involved in N-sulfation (black bars), 2-O- sulfation (gray bars), and 6-O-sulfation (striped bars). **(D)** Relative adherence differences of Gram-positive (●) and Gram-negative (○) bacteria to clones in which N-sulfation, 2-O-sulfation and 6-O-sulfation were silenced. The spreads represent the standard deviations. Statistically significant differences are denoted by ^**^, which indicates *p* < 0.01. We used RNA interference to silence the expression of the genes involved in these reactions.

To further establish the influence of sulfation at specific positions on the disaccharide unit of HS chains (Figure 6A), we used RNA interference to silence the expression of the genes involved in these reactions. Downregulation of N-sulfation of glucosamine residues was carried out by silencing NDST1, which reduced its expression with an efficiency of over 80%, and also reduced the expression of NDST2 about 70%, without affecting C5-glucuronate epimerization (GLCE), 2-O-sulfation (HS2ST1) or 6-O-sulfation (HS6ST1 and HS6ST2). Downregulation of 2-O-sulfation of uronic acid residues was performed by silencing HS2ST1, which reduced its expression with over 70% efficiency without affecting N-sulfation or 6-O-sulfation. Finally, downregulation of 6-O-sulfation of glucosamine residues was carried out by silencing HS6ST1, reducing its expression with an efficiency of over 80%, as well as the expression of HS6ST2 by around 70%, without N-sulfation or 2-O-sulfation being affected (Figure 6B).

Analysis of the adhesion of the pathogens to the knockout clones showed that the decrease in sulfation reduced the binding of all microorganisms tested. Interestingly, in all the cases, a decrease in N- or 6-O-sulfation, both of which involve the glucosamine residue, affected adherence to a greater extent than a reduction in 2-O sulfation, which involves the uronic acid residue (Figure 6C). When the effect on either Gram-positive or -negative bacteria was analyzed separately, N- and 6-O-sulfation varied considerably depending on the bacterium involved and differences between Gram type were not significant. In contrast, 2-O sulfation presented more homogenous results within each Gram type, with Gram-negative bacteria adherence being reduced by around 40% on average and Gram-positive by < 30%, a difference that was statistically significant (*p* < 0.01; Figure 6D).

## Discussion

The corneal epithelium is the eye's interface with the environment and, along with the other passive and active defense mechanisms, it acts as a barrier against pathogens. The initial attachment to host tissues is a critical event in pathogenesis, and a wide spectrum of bacterial pathogens, including *Helicobacter pylori, Bordetella pertussis, Mycobacterium tuberculosis*, and *Chlamydia trachomatis*, bind to cell surface PGs, and the majority of them have been shown to interact with their GAG moieties (Chen et al., 2008). These considerations make it of interest to analyze the involvement of corneal surface GAGs in adhesion and colonization by different bacterial pathogens.

Levels of GAG chains in cultured corneal epithelial cells were reduced either by enzymatic degradation with bacterial lyases or by the use of specific inhibitors of their biosynthesis. These treatments resulted in a significant decrease in bacterial adherence in all cases, indicating that GAG chains are involved in the attachment and colonization of bacteria. In addition, treatment with heparinases and chondroitinase together had an additive effect, suggesting the involvement of different species of GAGs in bacterial adherence. Interestingly, the effects observed in these experiments showed significant differences between Gram-positive and -negative bacteria, suggesting a differential use of GAGs as anchorage receptors for the two groups of microorganisms. This differential use of GAGs as receptor could be caused by the presence of different types of adhesins in the two bacterial groups, including different types of pili and other monomeric surface-bound adhesive proteins (Telford et al., 2006; Virji, 2008; Kline et al., 2009). It is also interesting to note that the inhibition of GAG synthesis resulted in significant and opposite effects on the adherence of Gram-positive and -negative bacteria, depending on the inhibitory molecule used: rhodamine strongly affected the binding of Gram-positive bacteria, while genistein produced a greater effect on Gram-negatives. Rhodamine B is thought to inhibit chain elongation, acting as a non-specific inhibitor that reduces GAG synthesis in a range of cells, and produces decreased lysosomal GAG storage in some types of mucopolysaccharidosis (Kaji et al., 1991; Roberts et al., 2006). The isoflavone genistein inhibits the kinase activity of epidermal growth factor receptors, which is required for full expression of genes coding for enzymes involved in GAG production (Piotrowska et al., 2006), although it has been described that the effect of this molecule on GAG biosynthesis is strongly dependent on their type and localization (Nikitovic et al., 2003). Accordingly, all of these data indicate that the corneal pathogens tested use GAGs, at least in part, as adhesion receptors, but they display two clearly distinct patterns of in the way they use these molecules, depending on their Gram nature.

The involvement of the predominant GAG species present on the cell surface in the adhesion of pathogens was studied by analyzing their ability to compete in adherence interference experiments. HS showed the highest inhibitory ability, suggesting that it constitutes the predominant cell surface receptor for all the bacteria analyzed. With the exception of *Streptococci*, CS B displayed the second highest inhibitory capacity, greater than that obtained for CS A and -C. These data become of particular relevance when considering that CS B is distinguished from the other CS species by the presence, in variable proportions, of iduronic acid, the C-5 epimer of glucuronic acid, which generates a more flexible polysaccharide chain, allowing specific interactions with several ligands, and that is also present in HS chains (Thelin et al., 2013).

In Gram-positive microorganisms, the use of a mixture of GAGs inhibited adhesion to the same extent as when using only HS. However, in Gram-negative bacteria, the use of a combination of GAGs increased inhibition by about 15% relative to HS, and this value was statistically significant, suggesting that in corneal epithelial cells HS is the main receptor for this group of microorganisms, but that other GAG species are also involved in a cooperative manner. The use of HS chains by different types of epithelial cells as receptors has been described in different pathogens, such as *H. pylori* (Ascencio et al., 1993), *Neisseria gonorrhoeae* (Chen et al., 1995a), or *S. aureus* (Hayashida et al., 2011), while others may use HS combined with other GAG species, as in the case of *Chlamydia trachomatis*, which binds to cervix-derived human epithelia through HS and CS B, but not CS A or -C (Zaretzky et al., 1995), and *S. pneumoniae*, which utilizes both HS and CS in the colonization of respiratory epithelial cells (Tonnaer et al., 2006). Interestingly, in some pathogens it has been described that different classes of GAGs mediate attachment to different types of mammalian cells, such as *Borrelia burgdorferi*, which uses HS to bind endothelial cells and CS B and HS together to glial cells (Leong et al., 1998), and *Chlamydophila pneumoniae*, which uses HS to bind epithelial cells but not lymphoid Jurkat cells (Kobayashi et al., 2011).

In addition, in all the cases analyzed the bacteria bound more efficiently to cells than to the ECM, a result analogous to those described for the adherence of other pathogens to different cell types, as in the case of *L. interrogans* (Breiner et al., 2009). Taken together, these data suggest that all the pathogens adhere principally to the corneal cells and not to the ECM, and that they predominantly use HS as receptor, although some Gram-negative bacteria seem to cooperatively use other GAGs.

HS is a complex linear biopolymer whose chains typically fall within the range 14–45 kDa (Lindahl and Kjellén, 2013). The length of the saccharide chain had a major effect on the binding; as expected, the high molecular weight chains inhibited the adherence of all pathogens similarly; however, the low molecular weight chains enhanced the inhibitory effect on Gram-negative microorganisms and, surprisingly, increased the adherence of Gram-positives by around 20%. Although the addition of HS to epithelial cells, which acts as a receptor, inhibits binding, there are cases where the opposite effect occurs, such as the adhesion of *Neisseria gonorrhoeae* to CHO cells, which is inhibited by high concentrations of heparin, but increased at low concentrations (Chen et al., 1995b). Furthermore, there are cases where the size of a polymer has a determining influence on binding to bacteria, such as with *L. interrogans*, which binds to high- but not to low-molecular-weight dextran sulfate (Breiner et al., 2009), and group B *Streptococcus*, in which GAG chain length is important to bind the surface-anchored Alpha C protein and promote bacterial entry into cervical epithelial cells *in vitro* (Chang et al., 2011). Moreover, a possible mechanism to explain the increase in adhesion of Gram-positive bacteria caused by low molecular weight HS, could be the formation of ternary complexes between bacterial adhesins, the HS and protein receptors of the cell surface, as has been proposed in the case of *N. gonorrhoeae* (van Putten and Paul, 1995) and *C. trachomatis* (Kim et al., 2011). As regards the increased inhibition observed in Gram-negative pathogens produced by low molecular weight HS, some cases have been described in which the size of the polymer greatly influences its inhibitory effect; for instance, the effect of the GAG acharan sulfate on the adhesion of *Helicobacter pylori* to Kato III cells (Sim et al., 2011).

Structurally, HS chains are composed of highly sulfated NS-domains interspaced with poorly sulfated NA-domains. The assay of both types of domains independently in adherence interference experiments showed that those that were highly sulfated had a greater inhibitory capacity than those poorly sulfated, and this effect was general and not dependent on the Gram nature of the pathogen. Although to our knowledge there are no previous data referring to HS domains, some published works have described that polymers that are more sulfated display greater inhibitory potential, such as *C. trachomatis, L. interrogans*, and group B *Streptococcus* (Zaretzky et al., 1995; Breiner et al., 2009; Chang et al., 2011). Not surprisingly, the interaction of HS with bacterial adhesins occurs to a greater extent through the highly sulfated domains, since these sequences are largely responsible for ligand binding, which depends to some extent, but not uniquely, on interactions which are mainly electrostatic in nature (Whitelock and Iozzo, 2005; Lindahl and Kjellén, 2013).

Consistent with the concept that the positioning of sulfate residues is critical for protein binding, most heparin binding proteins have clusters of basic amino acid residues that have been shown to be a requisite for heparin binding (Xu and Esko, 2014). Although there are many cases in which a single linear sequence does not define a HS-binding site, and two to three primary and secondary elements usually make up the binding site, it has been proposed that primary sequences composed of alternating basic and hydropathic residues are candidates for defining HS-binding motifs (Mulloy and Forster, 2000). We synthesized two peptides which included putative heparin binding sequences, QKKFKN and FKKKYGKS. Although both partially inhibited adhesion, the effect was particularly interesting for the short sequence, whose inhibition of Gram-negatives outweighed more than two-fold the values for Gram-positive, a statistically significant difference. These results suggest the involvement of proteins with different heparin binding sequences in both groups of bacteria, which could be related to the different nature of the adhesins described in the two microbial groups (Telford et al., 2006; Virji, 2008; Kline et al., 2009).

Generally, HS chains occur as glycoconjugates called proteoglycans (HSPGs). Two gene families, syndecans, and glypicans, account for most cell surface HSPGs, each of which consists of four and six isoforms, respectively (SDC1-4 and GPC1-6). Some previous studies have related specific species of cell surface HSPGs with adhesion and colonization by certain pathogens (van Putten and Paul, 1995; Chen et al., 2008; Hayashida et al., 2011). Since HSPGs are expressed in differing amounts depending on the tissue, we quantified their transcript levels in HCE-2 cells. We found evidence for the existence of transcripts of each of the four syndecan isoforms, but glypican levels were extremely low or undetectable, with the exception of GPC1. The experiments using enzymatic removal from the cell surface or blocking using specific antibodies concluded that syndecans are the main molecules involved in pathogen adherence to corneal epithelial cells, and that all the isoforms act in a cooperative way, independent of the Gram type of the bacteria. Although as far as we are aware this is the first time that a result of this type has been described, previous reports have pointed to the cooperation of several species of syndecans in pathogen binding, in particular links between SDC1 and SDC4 for *Listeria monocytogenes, Streptococcus pyogenes* and *Staphylococcal* sp., among others (Smith et al., 2006). Moreover, regarding the low participation of glypicans in the binding process, of particular interest is the case of *Chlamydophila pneumoniae*, which uses GAGs for adherence to epithelial cells, but whose adherence to lymphoid Jurkat cells, which only express GPC1, is carried out by a mechanism independent of GAGs (Kobayashi et al., 2011).

HS functions ultimately depend on the fine structure of the chains. Specific sets of variably modified disaccharides, usually within the sulfated domains, can define binding sites for a multitude of specific ligands. To investigate the influence sulfation at specific positions has on the disaccharide unit of HS chains, we used RNA interference to silence the expression of the genes involved in these reactions. The experiments have an added complexity due to, on the one hand, the existence of multiple isoforms responsible for some of these sulfations and, on the other, by the possible existence of enzyme complexes that are spatio-temporally regulated, both at the transcriptional and translational level (Victor et al., 2009). To overcome the problem of the isoforms, we selected lentiviral constructs which included sequences shared by all the genes currently known to be transcribed by HCE-2 cells. To verify that the specific sulfation studied in each case was downregulated, and that this fact did not affect the transcription of the epimerase and the rest of sulfotransferases, the transcript levels of all the genes involved were analyzed in each clone. Sulfation of the amino group is catalyzed by four different isoforms of N-deacetylase/N-sulfotransferases, NDST1-4; NDST3 and NDST4 are expressed primarily during embryonic development, but NDST1 and NDST2 show broad overlapping tissue distribution (Grobe et al., 2002), and the knockout of NDST1 effectively reduced the expression of both genes. HS2ST1 is a unique enzyme, but 6-O-sulfation is encoded by three genes, *HS6ST1*-*3*, and only transcription for isoforms one and two can be detected in HCE-2 cells, and our results showed that HS6ST1 silencing was also able to reduce the expression of HS6ST2. In all the cases, the knockout only affected the sulfation addressed.

Any reduction in sulfation affected the binding of pathogens, which is consistent with the data described above suggesting that these sulfated domains are largely responsible for bacterial adhesion. However, inhibition of adhesion was strongly influenced by the location of the sulfate group whose biosynthetic genes had been silenced. Binding was very sensitive to sulfation affecting the glucosamine residue, be it N- or 6-O-sulfation: In contrast, 2-O sulfation of the uronic acid residue had a lesser influence. Interestingly, the reduction in 2-O-sulfation was the only situation whose effects showed significant differences between Gram-positive and Gram-negative bacteria, resulting in greater inhibition of adhesion in the latter, results that indicate differences in interaction with the various adhesins from both microbial groups.

In this study, we used corneal cell cultures to reveal the involvement of GAGs in the adherence of pathogens to corneal cells. Cell cultures have been widely used as models to investigate pathogenesis, and some of these works are referred to in this article (Zaretzky et al., 1995; Chen et al., 1995b; van Putten and Paul, 1995; Leong et al., 1998; Tonnaer et al., 2006; Breiner et al., 2009; Chang et al., 2011; Kim et al., 2011; Kobayashi et al., 2011; Sim et al., 2011). This approach has proved valid, particularly for bacterial adherence, which constitutes the first event in pathogenesis. However, it must be taken into account that the situation *in vivo* displays many aspects which are not present in homogeneous cultured cells (Cowell et al., 1999; Hume et al., 2005). The complexities of the cornea, including variation in shape, and function of epithelial cells, the presence of other cell types, cell to cell communication or the immune system, points to interesting possibilities for future analysis of the data using animal models. Nevertheless, the molecular details described in this paper could provide a springboard for the development of new anti-infective strategies.

## Author contributions

BG, IA, and OG carried out the binding experiments. FV provided the bacterial clinical isolates. JA, BB, and AF contributed to cell cultures and data analysis. DR carried out the RNA interference experiments. JM and LQ co-ordinated the study and drafted the manuscript. All authors have read and approved the final manuscript.

### Conflict of interest statement

The authors declare that the research was conducted in the absence of any commercial or financial relationships that could be construed as a potential conflict of interest.
